# The Disruption of the Endothelial Barrier Contributes to Acute Lung Injury Induced by *Coxsackievirus* A2 Infection in Mice

**DOI:** 10.3390/ijms22189895

**Published:** 2021-09-13

**Authors:** Wangquan Ji, Qiang Hu, Mengdi Zhang, Chuwen Zhang, Chen Chen, Yujie Yan, Xue Zhang, Shuaiyin Chen, Ling Tao, Weiguo Zhang, Yuefei Jin, Guangcai Duan

**Affiliations:** 1 Department of Epidemiology, College of Public Health, Zhengzhou University, Zhengzhou 450001, China; ayq116814@gs.zzu.edu.cn (W.J.); huqiang011112@stu.zzu.edu.cn (Q.H.); zmd0208@stu.zzu.edu.cn (M.Z.); zcw@stu.stu.zzu.edu.cn (C.Z.); magical@stu.zzu.edu.cn (C.C.); yanyj9999@stu.zzu.edu.cn (Y.Y.); zx0108@stu.zzu.edu.cn (X.Z.); sychen@zzu.edu.cn (S.C.); 2 School of Public Health, Xinxiang Medical University, Xinxiang 453003, China; 141050@xxmu.edu.cn; 3 Department of Immunology, Duke University Medical Center, Durham, NC 27710, USA; wzhang033@icloud.com; 4 Henan Key Laboratory of Molecular Medicine, Zhengzhou University, Zhengzhou 450001, China

**Keywords:** hand, foot, and mouth disease, *Coxsackievirus* A2, endothelial barrier, pulmonary edema

## Abstract

Sporadic occurrences and outbreaks of hand, foot, and mouth disease (HFMD) caused by *Coxsackievirus* A2 (CVA2) have frequently reported worldwide recently, which pose a great challenge to public health. Epidemiological studies have suggested that the main cause of death in critical patients is pulmonary edema. However, the pathogenesis of this underlying comorbidity remains unclear. In this study, we utilized the 5-day-old BALB/c mouse model of lethal CVA2 infection to evaluate lung damage. We found that the permeability of lung microvascular was significantly increased after CVA2 infection. We also observed the direct infection and apoptosis of lung endothelial cells as well as the destruction of tight junctions between endothelial cells. CVA2 infection led to the degradation of tight junction proteins (e.g., ZO-1, claudin-5, and occludin). The gene transcription levels of von Willebrand factor (vWF), endothelin (ET), thrombomodulin (THBD), granular membrane protein 140 (GMP140), and intercellular cell adhesion molecule-1 (ICAM-1) related to endothelial dysfunction were all significantly increased. Additionally, CVA2 infection induced the increased expression of inflammatory cytokines (IL-6, IL-1β, and MCP-1) and the activation of p38 mitogen-activated protein kinase (MAPK). In conclusion, the disruption of the endothelial barrier contributes to acute lung injury induced by CVA2 infection; targeting p38-MAPK signaling may provide a therapeutic approach for pulmonary edema in critical infections of HFMD.

## 1. Introduction

Hand, foot, and mouth disease (HFMD) is a common acute infectious disease caused by several serotypes of *Enteroviruses* (EVs) including *Enterovirus* 71 (EV71), *Coxsackievirus* A16 (CVA16), or other types, and infants and children under 5 years of age are the major infected population [1]. Sporadic outbreaks of HFMD associated with *Coxsackievirus* A2 (CVA2) have been reported in many countries in recent years, such as China [2,3,4], Thailand [5], France [6], Brazil [7], and Korea [8], posing a great challenge to public health. To our knowledge, CVA2 infection is generally mild, but can sometimes develop into a severe case. It is worth noting that the conditions deteriorate sharply within a few hours to one day in some severe infections with symptoms such as dyspnea, coughing up pink-tinged, and foamy sputum indicating acute pulmonary edema. Epidemiological studies have suggested that pulmonary edema is the major cause of HFMD-related mortality [9]. In the past few decades, several mechanisms underlying the development of pulmonary edema have been proposed, one of which is neurogenic [10]. In detail, the sympathetic nerve is stimulated by brainstem encephalitis, leading to heart failure and pulmonary hypertension, and then pulmonary edema [11]. However, similar lung lesions are not observed in other severe encephalitis (such as Japanese encephalitis), and elevated blood pressure and pulmonary hypertension are not common in patients with pulmonary edema [12]. Our previous work and several studies have shown that inflammatory cytokines in the plasma or cerebrospinal fluid of HFMD patients with pulmonary edema are increased [13,14,15], and anti-inflammatory treatment has shown certain effects [16], emphasizing that systemic inflammation contributes to pulmonary edema. Pulmonary inflammation and increased vascular permeability resulting from systemic inflammation have been considered as an important cause of pulmonary edema. However, to date, the pathogenesis of pulmonary edema remains largely unclear. Therefore, it is necessary to study the mechanism of this underlying comorbidity and propose effective interventions to reduce the mortality of HFMD.

The integrity of vascular vessels is maintained by properly functioning endothelial cells, preventing fluid leakage [17]. The well-organized tight junctions (TJs) and adhesion junctions (AJs), the two most important connected components between endothelial cells, are lined on the vessel luminal face to maintain the integrity of the endothelial barrier [18,19,20]. Particularly, the TJs connecting endothelial cells in the pulmonary vasculature limit the transfer of fluid, proteins, and other constituents of blood to the alveolar spaces under normal conditions. A release of inflammatory mediators may lead to the open of endothelial barrier [21], permitting the passage of immune cells, antibody and complement molecules, as well as other substances from the bloodstream into the tissues, which may appear to play a major role in acute lung injury after CVA2 infection. A previous study demonstrated that EV71 and CVA16 can both break the epithelial barrier [22,23]. It is worth noting that VP1 of EV71 can damage the pulmonary epithelial barrier function via the downregulation of tight junction protein [24,25]. The contribution of lung endothelial barrier disruption to the pathogenesis of pulmonary edema has been largely neglected in previous studies. We speculate that CVA2 infection leads to the disruption of endothelial barrier and increases vascular permeability.

In this study, we applied an animal model of CVA2 infection-induced pulmonary edema to investigate the potential contribution to endothelial barrier, which will be helpful for understanding the pathogenesis of HFMD-associated pulmonary edema.

## 2. Results

### 2.1. CVA2 Infection Led to Acute Lung Injury in Mice

**Figure 1:**
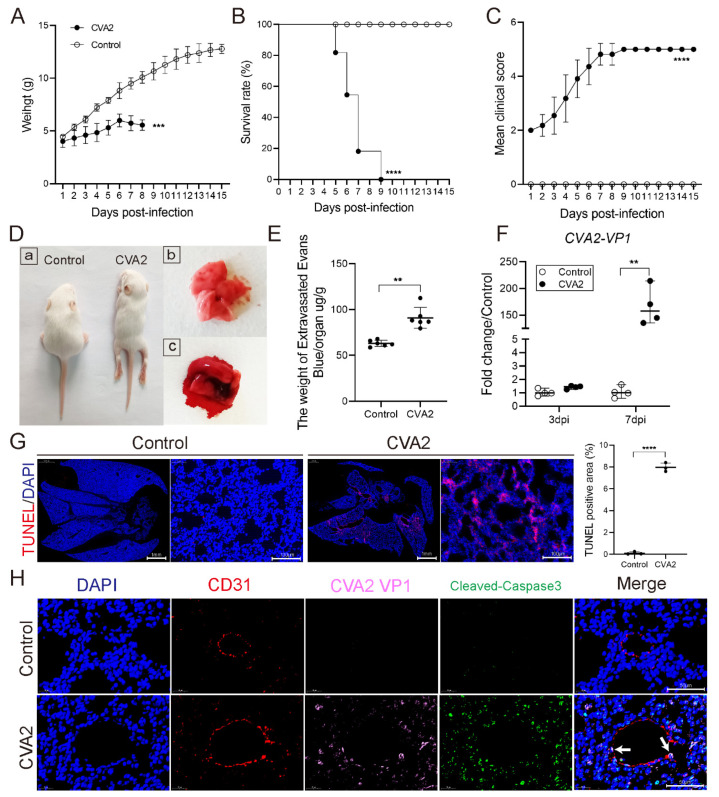
CVA2 infection led to acute lung injury in mice. (**A**–**C**) Body weights, survival rates, and mean clinical scores of mice in the experiments under optimized conditions (dosage (10^4^ TCID_50_/mouse), inoculation route (intramuscularly), and age (5-day-old BALB/c mice)) were monitored and recorded daily after inoculation with CVA2 until 15 dpi. Control animals were administered with equal culture medium (*n* = 10 per group). (**D**) Clinical manifestations of mice after CVA2 infection at 7 dpi. (**D**(**a**)): The representative clinical signs (weight loss, reduced mobility, ataxia, and single or double hind limb paralysis) induced by CVA2 infection in mice. The control mice treated with medium were healthy throughout the experiment. (**D**(**b**)): Normal lung tissue from control mouse. (**D**(**c**)): The lung tissue from CVA2 infected mouse (dark red with obvious bleeding and enlargement). (**E**) EB extravasation assay was performed to detect the pulmonary vascular permeability expressed as the ratio of EB content (µg) to the wet weight (g) of the lung tissues (*n* = 6 per group). (**F**) The relative expression of CVA2 VP1 mRNA was detected by qPCR (*n* = 4 per group). (**G**) TUNEL assay detected lung apoptosis at 7 dpi by immunofluorescence assay (*n* = 3 per group). (**H**) Colocalization of CD31, CVA2 VP1 and Cleaved-Caspase3 in lungs at 7 dpi was observed by immunofluorescence assay. The white arrows indicated cells with three proteins colocalization. Data represent the mean ± the standard error or median with range. ** *p* < 0.01; *** *p* < 0.001; **** *p* < 0.0001 vs. control. All experiments were repeated three times.

We utilized the CVA2 infection mouse model to evaluate lung damage. As shown in Figure 1A, the body weights of infected mice were obviously reduced compared to controls. All infected mice died between 5 and 9 dpi (Figure 1B), and the clinical scores (Figure 1C) of infected mice were significantly higher than that in controls. The picture (Figure 1D(a)) showed that the infected mice represented symptoms similar to infected human, such as weight loss, reduced mobility, and signs of neurologic disorder, such as limb paralysis. Control mice inoculated with culture supernatants of RD cells were healthy throughout the experiment. At 7 dpi, the control and infected mice were all euthanized and dissected. We found that the lungs of the infected mice were dark red, with obvious bleeding and enlargement (Figure 1D(c)), while the lungs of the control group appeared a normal pink color (Figure 1D(b)). Then, we detected the pulmonary vascular permeability using measuring the relative content of Evans blue (EB). The relative EB content in the lungs of infected mice was significantly higher than that in control mice (*p* < 0.01) (Figure 1E), implying an increase of vascular permeability after infection. To further evaluate lung damage caused by CVA2 infection, viral VP1 mRNA and apoptosis were measured. As shown in Figure 1F, CVA2 VP1 mRNA in the lungs of infected mice were much higher at 7 dpi, in comparison with control lungs. Additionally, CVA2 infection led to the increase of cell apoptosis, representing as TUNEL positive cells (Figure 1G), in the lungs of infected mice. Cells with colocalization of three proteins (CD31, CVA2 VP1, and Cleaved-Caspase3) could be detected in the infected mice (Figure 1H). Taken together, our findings suggest that CVA2 can directly infect pulmonary endothelial cells and result in acute lung injury (e.g., pulmonary edema, apoptosis) in mice.

### 2.2. Histopathological Changes of Lung Tissues after CVA2 Infection

**Figure 2:**
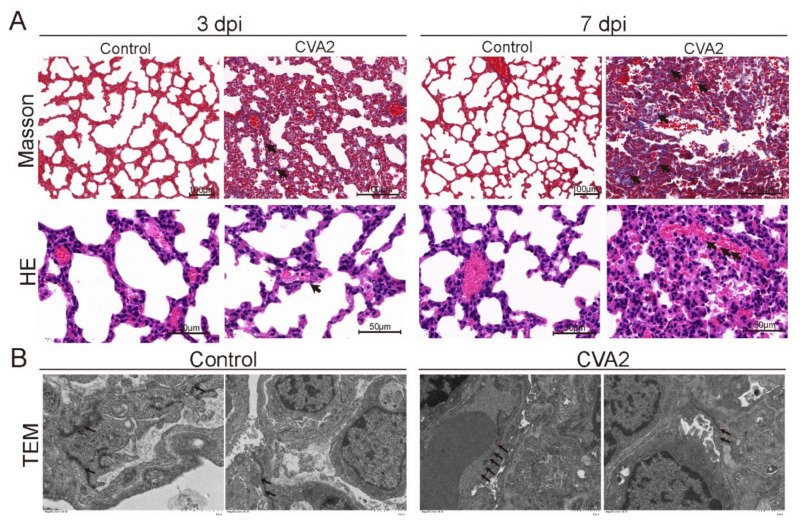
Histopathological changes of lung tissues after CVA2 infection. (**A**) Masson’s staining and HE were used to observe the pathological changes of lung tissue in mice at 3 dpi and 7 dpi. The black arrows indicate characteristic lesions. The alveoli collapsed, local pulmonary consolidation and diffused glial protein increase were observed in lungs of CVA2-infected mice. The infected lumens of alveoli were filled with edema fluid and red cells. No histological changes were observed in the lungs of uninfected mice. (**B**) The integrity of the pulmonary epithelium and morphological alterations of junctions was studied by TEM in both CVA2 infection and control groups. The arrows indicate tight junctions between endothelial cells. All experiments were repeated three times.

We performed Masson’s trichrome staining and HE staining (Figure 2A) in lung tissues at 3 dpi and 7 dpi to identify the pathological features of CVA2-infected mice. At 3 dpi, we observed thickened alveolar septum and collapsed alveoli in the lungs of the infected mice. At 7 dpi, vascular leakage and collagen deposition in alveolar space and the bronchi were observed in lung slices of infected mice, indicating congestion and inflammation. In control mice, the lungs were almost unchanged. The integrity of the pulmonary epithelium and morphological alterations of junctions were investigated by TEM in both groups. The control lungs showed intact junctions between the epithelial cells. In contrast, lung tissues from infected mice showed a loss of junctions between the adjacent pulmonary epithelial cells. The junctions were open and irregularly widened (Figure 2B). Collectively, CVA2 infection led to the disruption of the pulmonary epithelial barrier, and subsequently, vascular leakage.

### 2.3. The Degradation of Tight Junction Proteins in Lung Tissues after CVA2 Infection

**Figure 3:**
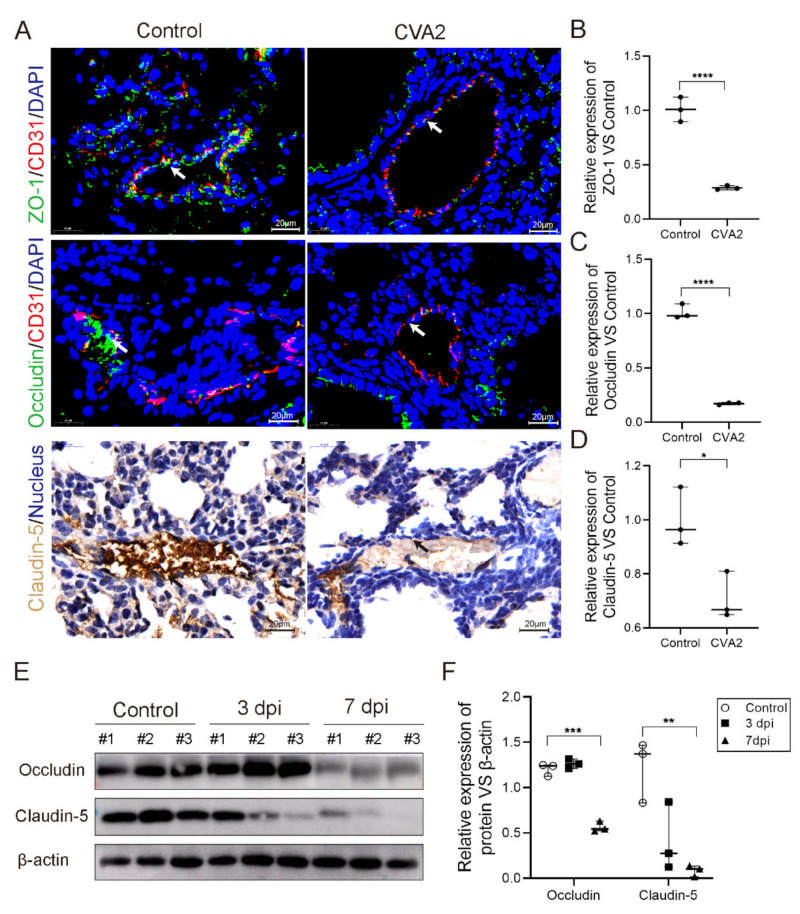
CVA2 infection led to the degradation of tight junction proteins in lung tissues. At 7 dpi, mice (*n* = 3 per group) were euthanized, and related proteins of isolated lungs were determined. (**A**) Immunofluorescence staining of ZO-1 (red); occludin (red) and immunohistochemical staining of claudin-5 were conducted. The arrows indicate the pulmonary microvessel. The relative expression levels of ZO-1 (**B**), occludin (**C**) and claudin-5 (**D**) compared to control group were calculated by Image J software. (**E**) The immunoblot bands of occludin and claudin-5. (**F**) The quantification of occludin and claudin-5. *n* = 3 per group, data represent median with range. * *p* < 0.01; ** *p* < 0.01; *** *p* < 0.001; **** *p* < 0.0001 vs. control. All the experiments were repeated at least three times.

Tight junction proteins are essential for maintaining endothelial permeability. To further assess the impact of CVA2 infection on the expressions of tight junction proteins, lung tissues from control mice and infected mice were subjected to immunofluorescence staining, immunohistochemical staining, and WB analysis at 7 dpi. As shown in Figure 3A, the expressions of ZO-1 (Figure 3B), occludin (Figure 3C), and claudin-5 (Figure 3D) located in the blood vessels were significantly downregulated in lung tissues from infected mice at 7 dpi, as quantified by average optical/fluorescence density. Consistent with above data, WB analysis showed a decrease of occludin in lung tissues of infected mice at 3 dpi and 7 dpi, and a downregulation in claudin-5 of lung tissues from infected mice at 3 dpi and 7 dpi (Figure 3E,F). Then, we measured the gene transcription level of the above proteins and found it did not change much at 3 dpi, but increased significantly at 7 dpi (Figure S1). Our data indicated the tight junction proteins were degraded in lung tissues after CVA2 infection.

### 2.4. The Effect of CVA2 Infection on Pulmonary Endothelial Function

**Figure 4:**
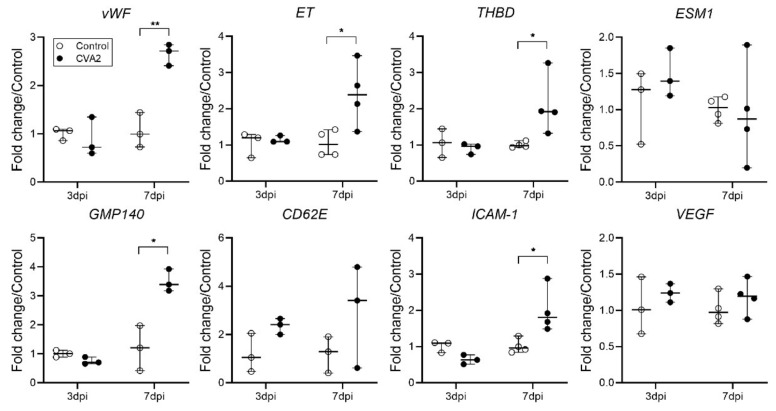
Effect of CVA2 infection on the mRNA levels of biomarkers of vascular endothelial cell dysfunction. qPCR was conducted to measure the expression levels of the related genes in lung tissues (3 dpi and 7 dpi) normalized to β-actin expression level. *n* = 3 or 4 per group, data represent median with range. * *p* < 0.05; ** *p* < 0.01 vs. control. All the experiments were repeated at least three times.

To determine whether CVA2 infection can affect endothelial function, the expressions of vWF, ET, THBD, ICAM-1, VEGF, ESM1, GMP140, and CD62E mRNA in lung tissues were assessed by quantitative-PCR (qPCR). Our results showed a sharp increase in vWF, ET, THBD, ICAM-1, GMP140, and CD62E mRNA levels in lung tissues of infected mice at 7 dpi, compared with controls. The levels of VEGF and ESM1 expression were slightly changed (Figure 4). These data suggested that CVA2 infection resulted in pulmonary endothelial dysfunction.

### 2.5. Monocytes Infiltration and Cytokines Expression Induced by CVA2 Infection

**Figure 5:**
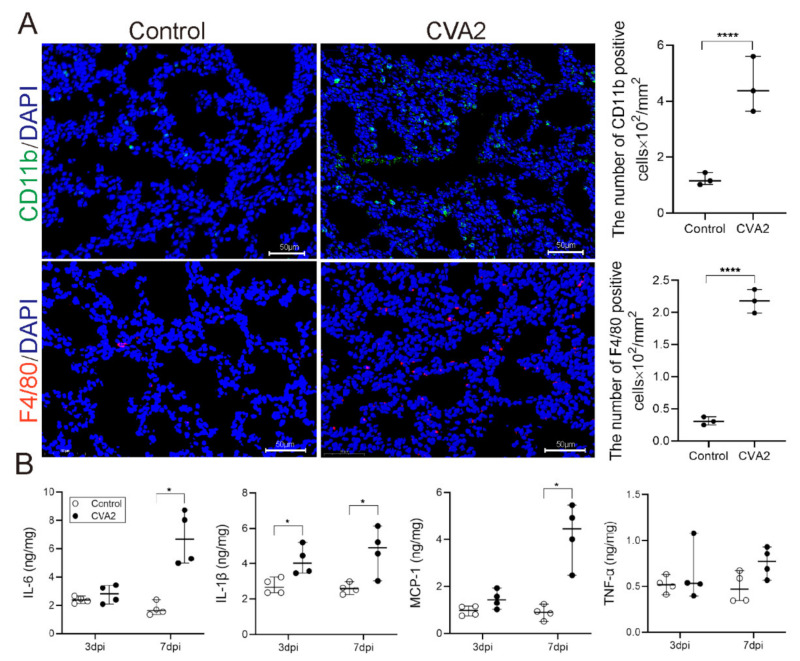
Inflammatory cell infiltration and cytokines expression induced by CVA2 infection. (**A**) CD11b and F4/80 positive cells in lung tissues were stained by IF and quantified by Image J software (*n* = 3 per group). (**B**) The expression levels of some cytokines (IL-6, IL-1β, MCP-1, and TNF-α) in tissue lysates of lungs by ELISA. *n* = 4 per group, data represent the median with range. * *p* < 0.05; **** *p* < 0.0001 vs. control. All the experiments were repeated at least three times.

To evaluate lung inflammation after CVA2 infection, the involved inflammatory cell and cytokines were determined. As shown in Figure 5A, CD11b positive cells (representing neutrophil, natural killer cell, macrophages, monocytes, and so on) in infected lung tissues were significantly higher than those in control mice, especially the macrophages (F4/80 positive cells). Subsequently, we detected the expression levels of IL-6, IL-1β, MCP-1, and TNF-α in tissue lysates by ELISA. At the earlier stage of CVA2 infection (3 dpi), the expression levels of IL-6, IL-1β, and MCP-1 were slightly increased with no statistical significance. When the disease developed into severity (7 dpi), the above three cytokines were significantly increased with statistical significance. However, there was no significant change in the expression of TNF-α at 3 dpi and 7 dpi (Figure 5B). Our data indicated that monocyte infiltration and proinflammatory cytokines contributed to the lung inflammation induced by CVA2 infection.

### 2.6. CVA2 Infection Activated p38-MAPK Signal Pathway in Lung Tissues

**Figure 6:**
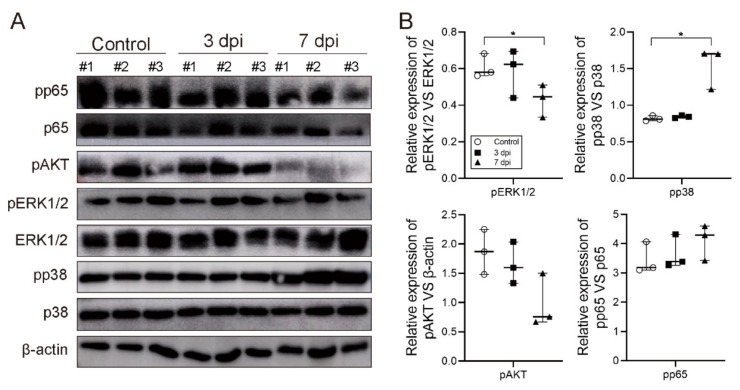
CVA2 infection activated p38-MAPK signal pathway in lung tissues. At 3 dpi and 7 dpi, control and infected mice were euthanized and isolated lung tissues for WB analysis. The immunoblot bands (**A**) and quantification (**B**) of related proteins in MAPK, NF-κB and PI3K/Akt signal pathways. pAKT was normalized by β-actin and others were normalized by p65, ERK1/2, or p38, respectively. *n* = 3 per group, data represent the median with range. * *p* < 0.05 vs. control. All the experiments were repeated at least three times.

To further explore the potential mechanism of CVA2-induced lung injury and inflammation, we determined the activation of the MAPK, NF-κB, and PI3K/Akt signal pathways. As shown in Figure 6A,B, except for the increase of pp38 (*p* < 0.05) and pp65, the expressions of pERK1/2, and pAKT all decreased at 7 dpi. Our results suggested that the p38-MAPK signal pathway is involved in lung injury and inflammation caused by CVA2 infection.

## 3. Discussion

Respiratory deterioration due to pulmonary edema and hemorrhage is a major cause of severe HFMD mortality [11]. However, so far, the mechanism of this severe lung injury has been fully investigated. Increasing evidence have suggested that enhancing vascular permeability contributes to acute lung injury after viral infection through endothelial dysfunction and local inflammation. In this study, we applied a CVA2 infection mouse model with acute lung injury and explored the possible mechanism of the disruption of endothelial barrier.

In the present study, infected mice showed pulmonary edema and hemorrhage, similar to human infections [26]. We detected the high expression level of CVA2 VP1 mRNA in lung tissues of infected mice and observed the direct infection of pulmonary endothelial cells, suggesting virus replication in the lung tissues. Meanwhile, we found that CVA2 infection dramatically increased lung cell apoptosis, including endothelial cells. The expressions of vWF, ET, THBD, ICAM-1, VEGF, ESM1, GMP140, and CD62E are associated with endothelial function. Our data showed that the mRNA expressions of vWF, ET, THBD, ICAM-1, and GMP140 were significantly increased, suggesting endothelial dysfunction [27,28,29,30]. Monolayer endothelial cells can regulate the transport properties for proteins, solutes, and fluid through paracellular and transcellular pathways [31]. The alveolar capillary membrane is critically dependent on endothelial barrier function to prevent pulmonary edema, and alterations or damage to the endothelial monolayer are sufficient to induce microvascular leak [31]. The pathological examination of lung tissues directly showed the decline of endothelial barrier. Therefore, our study suggests the disruption of endothelial barrier after CVA2 infection.

AJs and TJs, two morphologically distinct structures intermingling along the cell–cell junctional cleft, are essential components of the endothelial barrier [32,33,34]. TJs are characterized by promoting homophilic interaction between neighboring cells. The disruption of tight junctions could disturb the endothelial barrier and cause an increase in permeability. Claudins appear to be a major structural component of tight junctions, with claudin-5 being the predominant one among the various types of claudins expressed in endothelial tight junctions [35,36]. ZO-1 can be found ubiquitously within tight junction endothelial cells, which is a peripheral membrane-associated component of the cytoplasmic plaque of tight junctions [37]. In addition to claudins and ZO-1, occludin is a primary component of tight junctions [38]. The reduction of these tight junctions can result in vascular hyperpermeability [39,40]. We found that the gene transcription level of claudin-5, ZO-1, and occludin did not change much at 3 dpi, but increased significantly at 7 dpi. Therefore, our results suggested that the pathological feature of pulmonary edema, hemorrhage after CVA2 infection, was at least partly related to the degradation of tight junctions. We speculated that the loss of tight junctions may be caused by non-specific degradation, some proteases, such as matrix metalloproteases (MMPs) [41], or cell death through diverse pathways after CVA2 infection. MMPs are a family of zinc-dependent endo-proteases with multiple roles in tissue remodeling and the degradation of various proteins in the extracellular matrix [42]. In particular, MMP-2 and -9 may play an indispensable role in disrupting the endothelial barrier by damaging TJs [43,44]. At present, some studies have demonstrated that EV71 can invade the respiratory system of infected humans [45], and our animal model indeed showed viral replication and cell apoptosis in lung tissues of CVA2 infected mice. Therefore, the virus may directly lead to the apoptosis of endothelial cells, and subsequently, the degradation of tight junction proteins. Inflammatory stimulation may disturb pulmonary endothelial barrier homeostasis, resulting in increased permeability and protein-rich fluid extravasation [46]. The proinflammatory cytokines are critical for normal host defense, but they can damage the homeostatic protective functions of the normal endothelial barrier and potentiate pathological processes when produced inappropriately or in excess [46,47,48,49]. In recent years, many studies have been conducted on the cytokine levels related to pulmonary edema, suggesting that the important role of cytokines in the development of the pathogenesis of HFMD has been recognized. A significant elevation of TNF-α, IL-1β, IL-6, and MCP-1 levels of patients has been observed [50,51,52]. In this study, inflammatory cytokines (e.g., IL-6, IL-1β, MCP-1) were extremely higher in lung lysates of infected mice, which have also been detected in the serum of severe infections with HFMD. These cytokines may result in endothelial dysfunction and vascular leakage by transiently acting on the endothelium and altering the normal fluid barrier function of the endothelial cells [12]. The pulmonary endothelium contains a vast array of receptors that play important roles in regulating immune cells adherence, capillary permeability, and so on, all of which can be greatly altered by “cytokine storm” and lead to hemorrhage or edema [53,54]. Monocytes differentiate into macrophages and dendritic cells during migration into tissues [55]. The process of monocyte adhesion and migration across the endothelial cell layer is a key component of inflammatory responses. Pulmonary macrophages are large mononuclear phagocytic cells, which exert specific role in regulating the inflammatory response in lung injury [56]. Alterations in the balance and function of macrophage could lead to acute lung injury [57]. It was reported that the infiltration of pulmonary monocytes amplified local inflammation through cell adhesion molecules (ICAM-1) expressed by endothelial cells [58,59]. Monocyte infiltration can also release large amounts of cytokines, such as IL-6, IL-1β, and MCP-1. To further investigate the mechanism of lung damage and inflammation, we detected several important signal pathways. Our results suggest the involvement of p38-MAPK activation in acute lung injury by CVA2 infection. It is worth noting that p38 is a key signaling molecule in the regulation of proinflammatory cytokines and MMPs [60,61,62,63], which is consistent with our observations and assumptions above. Some studies have shown that the activation of p38-MAPK can cause endothelial dysfunction through a variety of pathways [64,65,66,67] and inhibiting the p38-MAPK can protect the endothelial barrier [68,69,70]. Therefore, blocking p38-MAPK provides a therapeutic approach for HFMD-associated pulmonary edema. We are conducting relevant experiments by using a p38 inhibitor in vivo to verify our hypothesis.

There are also some limitations in our study. Firstly, we used neonatal mice to establish the animal infection model of CVA2. It is known that the immune system of suckling mice is not fully developed. Secondly, we established the mouse model via intramuscular injection, which was different from the natural infection in humans. Lastly, some of the results reported here are based on 3–4 mice per group, which is a very small sample size. We recommend it should be cautious when extrapolating the conclusions of this study.

## 4. Materials and Methods

### 4.1. Cells and Viruses

Human rhabdomyosarcoma (RD) cells were cultured in Dulbecco’s modified Eagle’s medium (DMEM) (Thermo Fisher Scientific Inc., Waltham, MA, USA) supplemented with 10% fetal bovine serum (Thermo Fisher Scientific Inc., Waltham, MA, USA). The CVA2 strain used in the present study (HN202009, accession number: MT992622) was isolated from the stool specimen of a severely ill patient admitted to the First Affiliated Hospital of Xinxiang Medical University. The virus titers were determined by a 50% median tissue culture infective dose (TCID_50_) in accordance with the method of Reed and Muench [71].

### 4.2. Mice and Infection

The specific-pathogen-free (SPF) BALB/c mice used in this study were obtained from the Experimental Animal Center of Zhengzhou University (certificate no. DW2019110074), and housed in individually ventilated cages (IVC, Tecniplast, Milan, Italy) in a specific pathogen-free facility of the College of Public Health of Zhengzhou University on a 12 h light/dark cycle with ad libitum access to food and water. In the previous study, we introduced a neonatal mouse model of CVA2 infection based on 5-day-old BALB/c mice by i.m. inoculation (10^4^TCID_50_/mouse) [72]. The control mice were inoculated with an equal volume of culture supernatants of RD cells. The weights and clinical scores were recorded until 15 days post-infection (dpi). The grade of clinical disease was scored as described in previous study: 0, healthy; 1, lethargy and inactivity; 2, ataxic; 3, lose weight; 4, hind limb paralysis; 5, dying or death.

### 4.3. Detection of Pulmonary Vascular Permeability

Evans’s blue (EB) extravasation assay was performed as described previously [73,74]. EB dissolved in sterile phosphate-buffered saline was intraperitoneally (i.p.) injected into 5-day-old suckling mice at 6 dpi (20 mg/kg, *n* = 6 per group). Twenty-four hours later, mice lungs were taken out, washed with PBS, blotted dry, and weighed. Then, the lung tissues were homogenized in 1 mL of formamide, and the mixture was incubated at 60 °C in a water bath. One day later, the homogenate was centrifuged at 4000× *g*/min for 10 min at room temperature, and the absorbance of the supernatant was detected at 630 nm using a spectrophotometer. According to the optical density (OD) values of a series of different EB concentrations, we established the following formula: Y = 0.0694 × −0.0009, where x denotes EB concentration (µg/mL) and Y denotes the OD value. We used the above formula to calculate the pulmonary vascular endothelial permeability in lung tissues, expressed as the ratio of EB content (µg) to the wet weight (g) of the lung tissues.

### 4.4. Transmission Electron Microscopy

At 7 dpi, the lung tissues were harvested and diced into pieces approximately 1 mm^3^ each. Three or four pieces were immersed in the perfusion solution for 2 h for fixation, and then soaked overnight in a solution without glutaraldehyde before being washed in alkaline (0.03 mol/L of sodium hydroxide) sucrose (2%) solution. The specimens were then dehydrated applying a graded ethanol series. Next, the specimens were embedded in epoxy-resin embedding medium. Finally, ultrathin sections (90 nm), stained with uranyl acetate and lead citrate, were examined by using transmission electron microscopy (TEM) imaging (Hitachi, HT7800).

### 4.5. Histopathological and Immunohistochemical Analysis

At 7 dpi, control and infected mice were anesthetized with isoflurane and dissected, and then lung specimens were isolated and fixed in 10% paraformaldehyde for two days. After that, paraffin-embedded lungs were cut into 5 μm sections and stained with Masson’s trichrome for mucus secretion as well as hematoxylin-eosin (HE). The protein expression of claudin-5 in the lung specimens of control and infected mice was determined by immunohistochemical (IHC) staining in accordance with a standard immune-peroxidase procedure as described previously [75]. The images were captured by an inverted fluorescence microscope (OLYMPUS, IX73). The relative expression of claudin-5 was calculated using Image J software based on the percentage of positive area.

### 4.6. Immunofluorescent Staining

As mentioned above, after fixation, paraffin-embedded lungs were cut into 5 μm sections and the slices were stored at 4 °C until immunofluorescence staining was performed. For the immunofluorescence staining, technical services were provided by Servicebio Biotech Co., Ltd. (Wuhan, China) as described previously [76]. Images were captured using a fluorescence microscope (NIKON Eclipse CI, Tokyo, Japan) with the self-contained software (NIS-F-Ver43000-64bit-E) and the imaging system (NIKON Digital Sight DS-FI2, Tokyo, Japan). Fluorescence intensity was analyzed using Image J software.

### 4.7. Quantitative PCR

The cDNA was generated by using a reverse transcription kit (Yeasen Biotechnology (Shanghai) Co., Ltd., Shanghai, China) according to the manufacturer’s instructions after extracting total RNA from lung tissues using TRIzol reagent (Thermo Fisher Scientific Inc., Waltham, MA, USA). The gene transcription levels of biomarkers of endothelial dysfunction (von Willebrand Factor, vWF; endothelin, ET; Thrombomodulin, THBD; endothelial cell specific-1, ESM-1; granular membrane protein 140, GMP140; E-selectin, CD62E; intercellular cell adhesion molecule-1, ICAM-1; vascular endothelial growth factor, VEGF) and CVA2 VP1, ZO-1, Occludin, Claudin-5 were measured by quantitative-PCR (qPCR) (Yeasen Biotechnology (Shanghai) Co., Ltd., Shanghai, China) using the instrument (Serial No. q225-0207, Kubo Tech Co., Ltd., Beijing, China). The primers used for above experiments are listed in Table 1. All results calculated by the 2^−ΔΔCt^ method were normalized to the gene transcription levels of β-actin [77].

### 4.8. TUNEL Staining

To evaluate the damage of pulmonary cells after CVA2 infection, lung slices were stained with a TUNEL Apoptosis Assay Kit (Wuhan Servicebio Technology Co., Ltd., Wuhan, China) according to the manufacturer’s instructions.

### 4.9. Analysis of Tissue Lysates

At 7 dpi, whole lungs were removed, weighed, and quickly lysed in a cold isolation buffer (Beyotime Institute of Biotechnology, Shanghai, China) according to the manufacturer’s instructions. Next, according to the manufacturer’s instructions, using the BCA protein assay kit (Beijing Biomed Gene Technology Co., Ltd., Beijing, China), we measured the total protein concentration of tissue lysis fluid to standardize the following results. The expression levels of IL-6, IL-1β, TNF-α, and MCP-1 were measured using enzyme-linked immunosorbent assay (ELISA) kits (Biolegend, CA, USA).

### 4.10. Western Blotting

According to the manufacturer’s instruction, the total proteins of lung tissues were extracted using a protein extraction kit (CWbio Company Ltd., Beijing, China). After mixing with equal amount of 2 × sodium dodecyl sulfate (SDS) loading buffer, protein samples were separated by 10% polyacrylamide gel electrophoresis (PAGE) and then transferred to nitrocellulose (NC) membranes. After the NC membranes were taken from the electro transfer membrane system, it was blocked by 5% skim milk on the shaker at room temperature for at least 60 min and then incubated with primary antibodies. The NC membranes were washed thrice and incubated with corresponding secondary antibodies. After washing, the membranes were developed with an ECL enhanced Chemiluminescence Kit (Absin Bioscience, Inc., Shanghai, China) applying a professional instrument (Amersham Imager 600, General Electric Company, Boston, MA, USA). All the gray values (integrated density) of immunoblot bands were analyzed using Image J software to evaluate the expression levels of related proteins.

### 4.11. Antibodies

The following primary antibodies were used in this study: anti-ZO-1, occludin, pp65, p65, pERK1/2 (Thr202/Tyr204), ERK1/2, pp38, p38, pAKT (Thr308), anti-mouse IgG, anti-rabbit IgG (Cell Signaling Biotechnology, Inc., Danvers, MA, USA), anti-claudin-5, CD11b (Abcam Biotechnology, Inc., Cambridge, UK), anti-Cleaved-Caspase-3, CD31, F4/80 (Wuhan Servicebio Technology Co., Ltd., Wuhan, China), and anti-CVA2 VP1 (prepared in our own laboratory).

### 4.12. Statistical Analysis

Statistical analysis was performed with GraphPad Prism version 8.3 (GraphPad 8.3 Software, San Diego, CA, USA). The results were expressed as the mean ± standard deviation (SD) or median with range (non-normal distribution). The Mantel–Cox log rank test was used to compare the survival rates of different groups. Differences in the expression or transcription levels of relative protein were assessed using unpaired Student’s t-test or Mann–Whitney test (non-normal distribution). A *p*-value less than 0.05 was considered significant.

## 5. Conclusions

**Figure 7:**
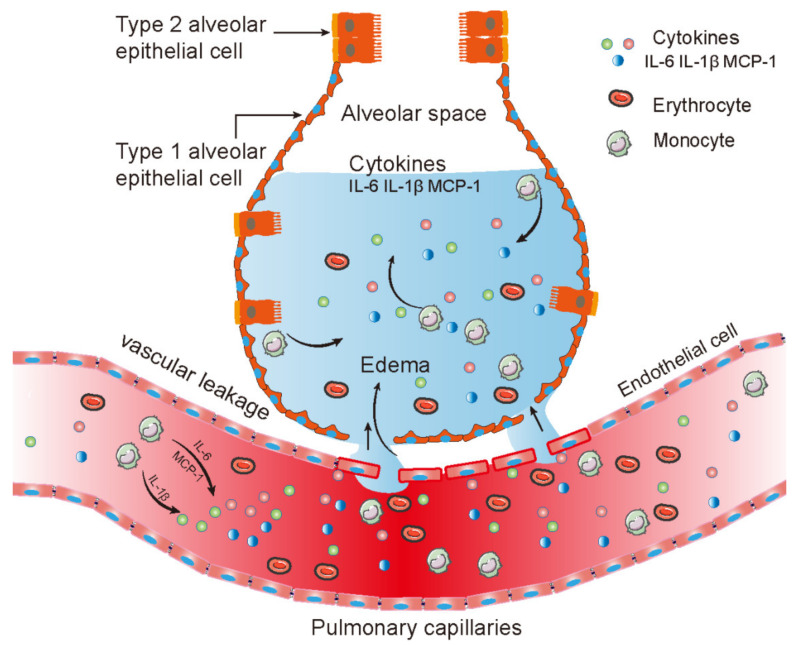
A hypothetical schematic of the pathogenesis.

In summary, our results suggest that the disruption of endothelial barrier contributes to pulmonary edema after CVA2 infection, (Figure 7), which may be caused either directly by the viral infection, or indirectly by endothelial dysfunction, the degradation of tight junctions, and inflammation.

## Figures and Tables

**Table 1:** Primers sequence.

Gene	Forward	Reverse	Product Lengths (bp)
CVA2-VP1	TCAGTCCCATTCATGTCGCC	AATGCGTTGTTGGGGCATTG	118
ESM1	CAAGTCTCTTTGCATTCCATCC	GGCTGAAGTGTCACTTTTACAG	106
VEGF	TAGAGTACATCTTCAAGCCGTC	CTTTCTTTGGTCTGCATTCACA	198
ICAM-1	CTGAAAGATGAGCTCGAGAGTG	AAACGAATACACGGTGATGGTA	141
vWF	GTGATTTTAACATCTTCGCGGA	CAGGAGTTGGCAAAATCATAGG	85
THBD	GCTGTGAGTACTTGTGCAATAG	TCACACATACAGGAGTAAGAGC	181
ET	TTTTCCCGTGATCTTCTCTCTG	CAGAAGTAGACACACTCCTTGT	192
GMP140	TGGGAGCAAGTGTGATAAGATG	GAACTGGCATGTGGATTTGTAG	161
CD62E	ACATTCACCGAGTTACTACTGG	GAGCCAGCTTCTTTTTGTTACA	218
ZO-1	CTGGTGAAGTCTCGGAAAAATG	CATCTCTTGCTGCCAAACTATC	97
Occludin	TGCTTCATCGCTTCCTTAGTAA	GGGTTCACTCCCATTATGTACA	155
Claudin-5	GTGGCACTCTTTGTTACCTTG	GATCATAGAACTCGCGGACAA	172
Mouse β-actin	GTGCTATGTTGCTCTAGACTTCG	ATGCCACAGGATTCCATACC	174

## Data Availability

All data generated or analyzed during this study are included in this published article and its Supplementary Information Files.

## Supplementary Materials

The following is available online at https://www.mdpi.com/article/10.3390/ijms22189895/s1.
