# Supplementary material for: The Disruption of the Endothelial Barrier Contributes to Acute Lung Injury Induced by Coxsackievirus A2 Infection in Mice
Source: Int J Mol Sci. 2021 Sep 13;22(18):9895. doi: 10.3390/ijms22189895 (PMC8467819; doi:10.3390/ijms22189895)
Supplement: Supplementary file 1 [file ijms-22-09895-s001.zip › ijms-1340073-supplementary.pdf]

Supplementary Material

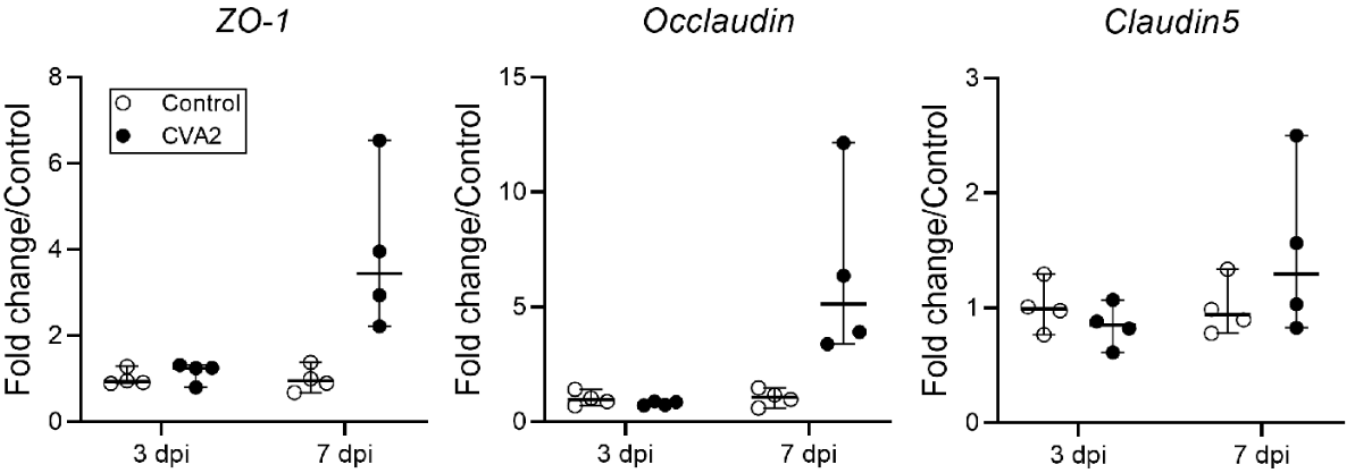

Figure S1. The gene transcription level of claudin-5, ZO-1, and occludin at 3 dpi and 7dpi in lung tissues normalized to β-actin expression levels (n = 4 per group). All the experiments were repeated at least three times.
